# Delayed diagnosis of polycythaemia vera in an adult female with non-cirrhotic portal hypertension

**DOI:** 10.4314/gmj.v56i1.6

**Published:** 2022-03

**Authors:** Kenneth Tachi, Victor Ekem, Yvonne Dei-Adomakoh

**Affiliations:** 1 Department of Medicine and Therapeutics, College of Health Sciences, University of Ghana, Accra, Ghana; 2 Department of Medicine and Therapeutics, Korle Bu Teaching Hospital, Accra, Ghana; 3 Department of Haematology, College of Health Sciences, University of Ghana, Accra, Ghana

**Keywords:** Polycythemia Vera, Diabetes Mellitus, JAK2, portal vein thrombosis, splenomegaly

## Abstract

Polycythaemia vera (PV) is a rare myeloproliferative neoplasm characterized primarily by erythrocytosis and an increased risk of thrombosis. We report a case of PV in a 60-year-old female with diabetes mellitus (DM) and a past history of recurrent abdominal pain and documented oesophageal varices who was followed up for 2 years as a case of non-cirrhotic portal hypertension of unknown cause. PV was only diagnosed after persistent complaints of vaso-motor symptoms and better scrutiny of full blood count results.

## Introduction

Polycythaemia vera (PV) is a chronic myeloproliferative neoplasm (MPN) characterized by excessive production of erythrocytes, and often leucocytes and platelets and JAK2 mutations (V617F or exon 12) in most cases. The 2016 revised WHO guidelines for PV diagnosis consist of three major criteria and a minor criterion. The major criteria are 1. Haemoglobin (Hb) >16.5g/dl or Haematocrit (Hct) >49% for men or Hb> 16g/dl or Hct > 48% for women 2. Bone marrow trilineage proliferation with pleomorphic mature megakaryocytes and 3. The presence of JAK 2 V617F mutation, whiles the minor criterion is subnormal serum erythropoietin level. Either all three major criteria **OR** major criteria **1** and **2** and the minor criterion must be present to make a diagnosis PV.^1^

The clinical features of PV arise mainly from hyperviscosity, which may cause thrombosis. Subsequent progression to myelofibrosis and leukaemic transformation may also cause symptoms. PV should be suspected in any patient with elevated Hb/hct, splenomegaly and/or portal hypertension with or without gastro-oesophageal varices. Hypersplenism in these patients may mask the erythrocytosis and thus delay the diagnosis.^2^ We present a case of a Ghanaian adult female diagnosed with oesophageal varices, the cause of which remained elusive until thorough scrutiny of her previous full blood count (FBC) results.

## Case Report

We present a 60-year-old Ghanaian female diabetic patient regularly followed up at the Diabetic Unit of the Korle Bu Teaching Hospital in Accra, Ghana.

The patient was referred to the outpatient unit of the Surgical Department of the Korle-Bu Teaching Hospital from a private clinic in 2015 with a four-year history of recurrent abdominal pain suspected to be due to a paraumbilical hernia. She reported an upper GI endoscopy done in April 2010 to evaluate abdominal discomfort at the time. It showed *H. pylori* negative gastroduodenitis and oesophageal varices then. Her symptoms did not significantly improve despite prolonged proton pump inhibitor therapy. At the Surgical Department, the surgeons documented a reducible, non-obstructed paraumbilical hernia and splenomegaly but concluded these were not the cause of her abdominal symptoms. She was subsequently referred to the gastrointestinal (GI) clinic of the hospital with a diagnosis of portal hypertension.

Further clinical evaluation at the GI clinic did not reveal any new findings. Specifically, she denied alcohol use and had no stigmata of chronic liver disease. Her liver function test was normal, and Hepatitis B and C screens were also negative. She also underwent a colonoscopy as part of the evaluation of her abdominal pain but the findings were normal. Her initial FBC results are shown in Table 1.

**Table 1:** Hb, Hct, WCC and platelet levels prior to diagnosis, at diagnosis and after initiation of treatment

	First attendance	Pre-diagnosis		Diagnosis	After initial treatment		Most recent	
**DATE**	12/6/15	10/8/16	16/3/2017	10/4/17	24/5/17	21/6/17	18/7/18	14/11/18
**HB (g/dl)**	15.4	17.7	18.0	18.2	15.6	13.3	13.2	14.9
**RBC(x 10^9^/l)**	5.88	6.40	6.49	6.55	6.22	5.34	3.99	4.89
**HCT (%)**	49.1	53.2	55.1	54.1	48.5	42.1	39.2	45.7
**WBC(x 10^9^/l)**	8.2	22.52	13.6	14.5	4.7	2.4	4.7	8.1
**Platelets (x 10^9^)**	580	670	580	518	478	137	267	318

A repeat upper GI endoscopy done reported normal gastric and duodenal mucosae and confirmed the oesophageal varices, which were then banded. A diagnosis of oesophageal varices from non-cirrhotic portal hypertension of undetermined cause was made and she was started on propranolol for primary variceal prophylaxis, but she was lost to follow up at the GI clinic thereafter.

In March 2017, she was referred again by her family physician to the surgeons on account of constant severe abdominal pain radiating to her back and associated anorexia, nausea and vomiting that had persisted for 3 weeks. Examination at the time showed massive splenomegaly. Abdominal ultrasound and CT scans both confirmed the presence of massive splenomegaly. There was however no comment on the portal vein. Her pain was managed conservatively, and she was discharged home on day 4 to follow up at the GI clinic.

**Figure 1:**
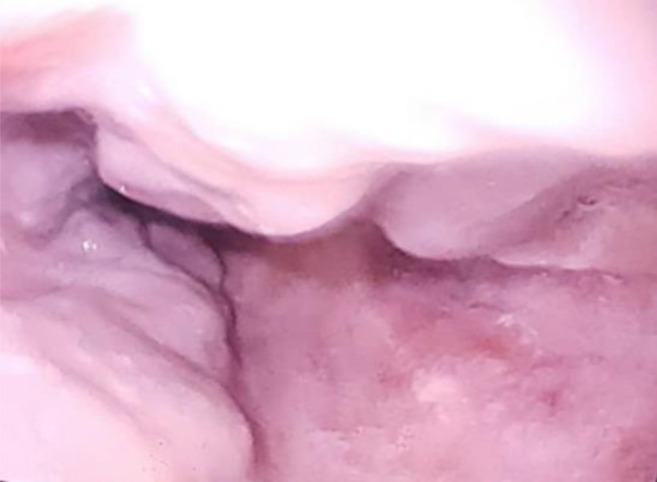
Columns of oesophageal varices seen at endoscopy

She visited the GI clinic in March 2017 following her second referral, and was re-evaluated with no new findings or conclusions. She had a repeat upper endoscopy that showed varices (Figure 1) and variceal banding was again done. She was restarted on propranolol but was again lost to follow up at the clinic.

Meanwhile, she continued to visit the diabetic clinic with recurrent complaints of headaches and palpitations. Careful examination during one such visits in April 2017, revealed a dark, ruddy complexion and plethora of the conjunctivae.

An FBC report (shown in Table 1) revealed an unusually high Hb (18g/dL) and Hct (55.1%). A review of a series of FBCs done in 2016 showed a similar trend (see Table 1). A diagnosis of PV was suspected at this stage and further evaluation including JAK2 mutation analysis and a bone marrow biopsy was undertaken to confirm same.

**Figure 2:**
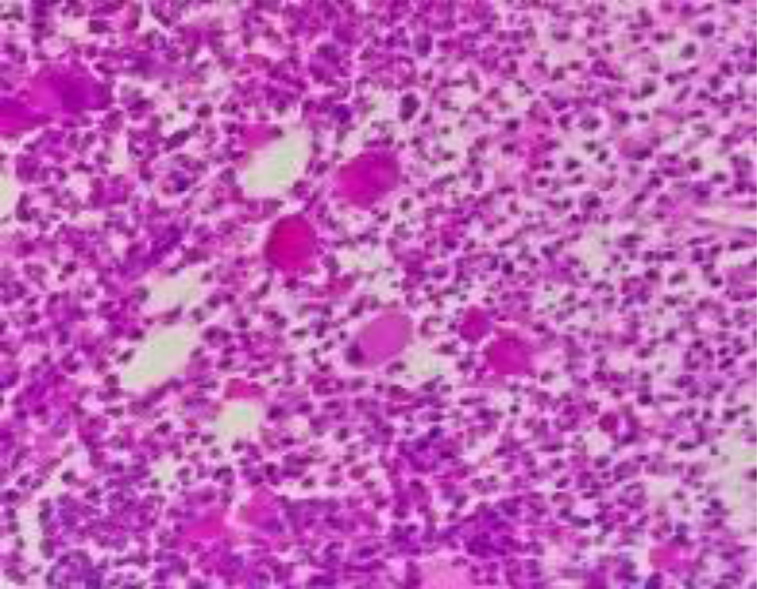
Section of bone marrow trephine biopsy showing increased cellularity with an increase in megakaryocytes and mononuclear megakaryocytes

She was reviewed by the haematology team when the results of the JAK2V617F gene mutation returned positive. A bone marrow trephine biopsy also confirmed a hypercellular marrow with trilineage haemopoiesis and abnormal megakaryocytes consistent with MPN. (Figure 2).

The bone biopsy also helped differentiate early PV with concomitant thrombocytosis from essential thrombocythemia. Her serum erythropoietin level was also subsequently noted to be low. These results confirmed PV. Karyotyping was, however not done to assess prognosis. Concurrently, she was re-evaluated by the gastroenterologist for the cause of her non-cirrhotic portal hypertension.

Re-evaluation with ultrasound, doppler and abdominal CT scan with a specific request for comments on the portal vein was made.

The radiologist, on these occasions, reported a normal liver and chronic portal vein thrombosis, portal hypertension with splenomegaly and portosystemic shunts. A diagnosis of portal hypertension from portal vein thrombosis secondary to PV was made in May 2017, two years after the initial presentation of varices.

The patient was started on hydroxycarbamide and aspirin. She also had multiple sessions of venesection. She has continued propranolol for primary variceal prophylaxis and lisinopril and metformin for hypertension and diabetes respectively. Except for an initial diarrhoea after the start of the hydroxycarbamide, she has been well. Her headaches, dizziness and abdominal pain have all subsided. Her blood counts have progressively normalized as shown in Table 1.

## Discussion

Polycythaemia vera is a chronic MPN characterized by JAK 2 mutations; JAK2V617F in 95% of PV patients and JAK 2exon 12 in another 2–4% of cases. 3 In a 2013 large international study of 1545 patients, the median age for PV was 61 years and the male: female distribution was close to 1:1. 3

The principal clinical features of PV are related to the increased cell mass causing hyperviscosity and, therefore, thrombosis. All organ systems can be affected by thrombosis, and the presence of the JAK2V617F mutation is considered a risk factor for thrombosis.^4^ PV patients present with vasomotor symptoms in 30% of cases.^3^ These include headache, lightheadedness, dizziness and palpitations as occurred in our patient and are thought to be due to small-vessel occlusion by thrombi. About 40% of PV patients also report aquagenic pruritus, the presence of which is associated with a lower risk of arterial thrombosis.^5^, ^6^

Palpable splenomegaly as in the index case, has also been reported in 1/3 of PV patients in Malaysia.^7^ Whiles Hb > 16 and 16.5 in women and men respectively should raise suspicion for PV diagnosis, the presence of splenomegaly can mask high Hb and delay diagnosis. Even though this did not occur in our case, clinicians must be mindful of this fact when interpreting Hb results as part of the evaluation for PV. In this case, the initial Hb of 15g/dl was high for the typical Ghanaian woman presenting to the clinic, but not high enough to warrant an investigation for PV, save for a high index of suspicion. However, abnormally high Hb and Hct recorded in subsequent clinic attendances in August 2016 and March 2017 should have alerted the attending clinicians to the possibility of PV. It is likely that because FBC is often considered ‘routine’ by many clinicians, the scrutiny for abnormalities may not have been thorough.

If delays in diagnosis such as occurred in this case are to be avoided, there must be a paradigm shift towards greater scrutiny of every test ordered by clinicians and seeking answers for any detected abnormalities.

PV can be complicated by portal vein thrombosis (PVT).^8^ In the acute phase, PVT may present with abdominal pain, nausea, vomiting and fever. On hindsight, it is possible that our patient's acute abdomen presentation was an episode of acute PVT that was missed. Chronic portal vein thrombosis from PV may be characterized by clinical features of portal hypertension including oesophageal varices.^2^, ^9^, ^10^ From our patient's medical records, she must have had PV predating April 2010 when the first upper GI endoscopy confirmed oesophageal varices. Even though portal hypertension was identified then, a purposeful search for portal vein thrombosis was not undertaken. Had that been done, an earlier evaluation for PV with JAK2V617F screening might have been undertaken. A meta-analysis of JAK2V617F screening in splanchnic vein thrombosis patients without typical haematological MPN features identified MPN in 15.4% of patients with PVT.^11^

To effectively manage PV, it is important to risk stratify the PV patient to help estimate the risk of recurrent thrombosis. Accordingly, PV includes two risk categories: high-risk (age >60 years or thrombosis history) and low-risk (absence of both risk factors).^12^ All patients with PV require phlebotomy to keep haematocrit below 45% and once-daily aspirin 81 mg to prevent thrombotic complications. In addition, high-risk patients with PV require cytoreductive therapy with hydroxycarbamide as the first-line drug.^12^–^14^ Our patient thus was in the high risk category and received the standard of care with good outcomes. Treatment with ruxolutinib or other JAK2 inhibitors is reserved for patients who fail the aforementioned or patients with severe pruritus or massive splenomegaly.^15^ The prognosis of PV is worse than for the general population with a reported median survival of 14years from some studies.^3^, ^16^ It remains whether early diagnosis and treatment confers any prognostic advantage.

## Conclusion

In conclusion, we have presented a case of 60-year-old Ghanaian whose diagnosis of PV was delayed for 7 years because of the protean presentation and the inertia in evaluation for the cause of her portal hypertension and a failure to scrutinize multiple FBC results thoroughly. PV should be considered in the differential diagnosis of portal hypertension of unknown cause, and the identification of PVT should prompt a JAK2V617F screening and workup for PV.
